# Dietary Behaviour and Nutrition in Patients with COPD Treated with Long-Term Oxygen Therapy

**DOI:** 10.3390/ijerph182312793

**Published:** 2021-12-04

**Authors:** Dominika Mekal, Aleksandra Czerw, Andrzej Deptala

**Affiliations:** 1 Department of Cancer Prevention, Medical University of Warsaw, ul. Zwirki i Wigury 81, 02-291 Warszawa, Poland; andrzej.deptala@wum.edu.pl; 2 Department of Health Economics and Medical Law, Medical University of Warsaw, ul. Zwirki i Wigury 81, 02-291 Warszawa, Poland; aleksandra.czerw@wum.edu.pl

**Keywords:** chronic obstructive pulmonary disease, long-term oxygen therapy, diet, nutritional behaviour, respiratory system function, nutritional assessment

## Abstract

Background: It is the first study in Poland and one of the first in the world to assess the nutrition of patients with chronic obstructive pulmonary disease (COPD) treated with long-term oxygen therapy (LTOT). Methods: The study group consisted of 110 COPD patients treated with LTOT. Anthropometric measurements and spirometry were performed. The diet of patients was assessed using a 3-day nutrition diary. Results: When assessing the degree of airflow obstruction (FEV1% N) depending on the BMI in patients treated with LTOT, a statistically significant correlation was demonstrated between the BMI and the value of the FEV% N parameter (*p* = 0.0093). Patients with COPD with a BMI >30 had statistically significantly higher values of FEV1% N than patients with a BMI in the range of 20–24.9 (*p* = 0.0278). Intake of calcium, vitamins A, C, D, E and folates was lower than the recommended daily intake in more than 95% of COPD patients. Conclusions: The diet of COPD patients treated with long-term oxygen therapy was improperly balanced, with deficiencies of important nutrients. Airflow obstruction in the respiratory tract was significantly smaller in obese patients, and greater in patients with diagnosed malnutrition.

## 1. Introduction

Increasingly, the method and quality of nutrition constitute important factors that have a significant impact on the occurrence, course and effectiveness of treatment. According to studies, 95% of COPD patients are ex-smokers, but only about 20% of ex-smokers develop COPD [1]. This means that other factors, e.g., nutritional status, may constitute a poor prognostic factor in patients diagnosed with COPD. Other factors may also protect against the development of the disease or contribute to its development [2]. The risk of COPD may be lower in people maintaining a proper diet [3]. A proper diet is also crucial for maintaining proper health in COPD patients, for whom a diet rich in antioxidants is associated with improved lung function and reduced long-term mortality [4,5]. Epidemiological evidence suggests that in COPD, nutrition may be associated with the risk of disease progression [6]. Therefore, studying the diet of COPD patients in the Polish population is clinically very important. Identification of modifiable risk factors for the prevention and treatment of COPD is urgent, and the scientific community has begun to pay particular attention to diet as an integral part of COPD management, from prevention to treatment. Therefore, a better understanding of the effects of diet on the prevention and/or results of COPD can increase scientific and clinical awareness of the importance of nutritional approaches as well as provide guidance for future research and strategies for promoting pulmonary health and preventing the onset and progression of the disease. Therefore, in this study, the main objective was to evaluate the diet of COPD patients treated with long-term oxygen therapy.

Although definitive data is lacking, the available scientific evidence suggests that some foods and nutrients, especially nutraceuticals with antioxidant and anti-inflammatory properties, are associated with lung function, less frequent lung function failure, and may prevent disease progression [7]. Our study also attempted to evaluate the consumption of individual nutrients and their effect on lung function.

Antioxidants, such as vitamin E, C, beta-carotene, ubiquinone, flavonoids, quercetin and selenium are found in food products and are considered the “first line of defence” against free radicals that can be harmful to cells and contribute to inflammation [8]. The imbalance between oxidative stress and antioxidants is a major risk factor in the pathogenesis of some chronic diseases, i.e., COPD [8]. One of the assumptions of the diet of COPD patients is the reduction of chronic metabolic stress, which may be an effective therapeutic strategy in these patients. Many studies have showed that high consumption of vegetables and fruit is associated with a lower risk of COPD [3,9,10,11]. Large cohort studies from Singapore have shown that frequent consumption of fruit (i.e., apples, pears, grapes) can reduce the incidence of expectorant cough. It has been suggested that flavonoids found in vegetables and fruits act as an anti-inflammatory factor in the lungs, and frequent consumption of antioxidant-rich foods is associated with improved lung function. The protective effect of vegetables and fruits may also be associated with the presence of high doses of vitamin A, which has been recognized as a vitamin that reduces the risk of COPD [3]. 

In the large population-based Atherosclerosis Risk study of 11,897 American women and men aged 44–66 years, higher total dietary fibre intake was associated with better lung function in spirometry [12]. 

Studies conducted in a representative group of US residents, aged 45 or older, have shown that frequent consumption of cured meat was associated with lower values of FEV1, FEV1/FVC (forced vital capacity) and increased risk of COPD. In people who ate cured meats more often than other food products, a lower consumption of vitamin C and beta-carotene was observed [13]. Dietary recommendations for patients at risk of developing COPD are consistent with general dietary recommendations. A diet low in fat, high in dietary fibre, rich in complex carbohydrates, including large amounts of vegetables and fruits, is recommended. Dietary recommendations in patients with advanced COPD should be determined individually for each patient, taking into account the degree of malnutrition, as well as the socio-economic conditions of individual patients. 

Chronic obstructive pulmonary disease is currently recognized as a disease with significant systemic consequences that influence its course and prognosis. One of the most common extrapulmonary symptoms is poor nutritional status and muscle weakness. Maintaining the proper nutritional status in patients with respiratory diseases is important because malnutrition directly affects the function of the lungs and respiratory tract, as well as the respiratory muscles and lung parenchyma [14]. Malnutrition is a common and often underestimated problem in patients with COPD. In the Copenhagen City Heart Study conducted by Landbo et al., it was found that the BMI index is an independent predictor of mortality in patients with COPD [15]. It has also been found that patients with COPD with a BMI below 20 kg/m^2^ are at greater risk of exacerbations compared to patients with a BMI of 20 kg/m^2^ [16,17].

The causes of malnutrition in COPD are believed to be multifactorial and include tissue hypoxia, decreased muscle mass, increased resting metabolic rate, systemic inflammation, oxidative stress, the effects of certain drugs (corticosteroids), hypogonadism, and the advantage of catabolism over anabolism [18]. The mechanisms of development of these disorders have not been fully understood yet. In the study group, body weight was assessed using the BMI index. Spirometry was performed to evaluate parameters assessing lung function depending on the BMI index. 

Considering the alarmingly increasing burden of COPD worldwide, the identification of modifiable risk factors in the prevention and treatment of COPD is highly desirable. Based on the available evidence, greater awareness of diet and dietary factors, influencing respiratory health, may be of interest for public health due to their disease modifying effects. Many studies in the general population and in people with respiratory diseases report that current eating habits are qualitatively poor and therefore leave a lot of room for improvement and intervention.

This study is aimed at assessing diet of COPD patients treated with long-term oxygen therapy. It is the first study in Poland to assess the nutrition of COPD patients treated with long-term oxygen therapy (LTOT) and one of the first studies in the world.

## 2. Materials and Methods

In the study, 110 patients (39 women and 71 men) participated; mean age 68.4 ± 8.9 years. They were treated with long-term oxygen therapy. The study was carried out from July 2015 to January 2016 in patients who were diagnosed and treated: in the 1st Department of Lung Diseases and Respiratory Allergy of the Voivodeship Healthcare Complex and the Centre for Treatment of Lung Diseases and Rehabilitation in Łodz, in Med-Med Specialist Non-Public Healthcare Institute in Łodz, at the Medical Centre Lucyna and Andrzej Dymek in Zawadzkie, and Strzelce Opolskie. The consent of the heads of the units was obtained for carrying out tests in the above-mentioned centres. This study included patients who gave informed, written consent and met the following inclusion criteria: clinical diagnosis of COPD based on the 2015 Global Initiative for Chronic Obstructive Lung Disease (COPD) guidelines [19] and demonstrated a stable clinical condition without a past COPD exacerbation in the last 3 months. The exclusion criteria were: diagnosed neoplastic process, dementia, epilepsy, permanently implanted heart stimulation system, severe mental illnesses and failure to sign informed consent. 

The assessment of nutrition among COPD patients treated with long-term oxygen therapy was carried out using a 3-day nutrition diary. A pilot study was conducted.

### 2.1. Three-Day Nutrition Diary, Quantitative Dietary Assessment

Patients’ diet was assessed on the basis of a 3-day nutrition diary filled in by the study participants. The study participants were instructed in detail on how to fill in the diary.

The quantity of products and drinks consumed by the study participants was determined in home measures, which were then converted into weight units based on the “Products and dishes photo album” [20]. The nutritional value was calculated with the use of the computer program “Diet 5” (Program manufacturer: National Food and Nutrition Institute), using tables of the composition and nutritional value of products [21], taking into account the loss of vitamins. A consumption data analysis has been performed by a clinical dietician. Total energy (kcal), protein (g, %), fat (g, %), carbohydrates (g, %), sucrose (g), lactose (g), dietary fibre (g), sodium (mg), potassium (mg), calcium (mg), phosphorus (mg), magnesium (mg), iron (mg), zinc (mg), copper (mg), vitamin A (μg), vitamin E (mg), vitamin D (µg), vitamin B1 (mg), vitamin B2 (mg), vitamin B6 (mg), folate (µg), vitamin B12 (µg) and vitamin C (mg) were calculated based on 3 days of oral nutrition.

The composition of dietary nutrients was expressed as a percentage of the Recommended Dietary Allowance (RDA), Appropriate Intake (AI) and Tolerable Upper Intake Level (UL) for adult age/gender groups of the Polish population according to the Nutrition Standards of the Food and Nutrition Institute [22]. Values being ±10% of the norms recommended by the Polish Food and Nutrition Institute were considered normal values, obtained from the calculations of all nutrients [22].

### 2.2. Anthropometry

In all study participants, body weight, expressed in kilograms, was measured using a certified scale, and body height, expressed in centimetres, was measured using a certified stadiometer. Height and weight were measured with accuracy to cm/kg.

Based on the obtained results of height and weight measurements, the Body Mass Index (BMI) was calculated using the standard formula. Based on the analysis of literature [15,23,24,25,26,27] on the nutritional status of COPD patients and in accordance with the meta-analysis performed by Chao et al. [28], BMI = 20 kg/m^2^ was adopted as the limit of malnutrition. 

Details of measurements and calculations are provided in Appendix A.

### 2.3. Spirometry

Respiratory function tests were performed in the morning using a Care Fusion Micro Lab UK spirometer according to the recommendations of the European Respiratory Society [19]. FEV1/FVC (after inhalation of the bronchodilator) <0.70 was assumed as the criterion of bronchial obstruction. The MEF value of 50 (L/s) and (% of normal ranges) was also measured [19].

### 2.4. St. George Questionnaire (SGRQ-C) to Assess the Quality of Life in COPD Patients

The St. George Questionnaire (SGRQ-C—St. George’s Respiratory Questionnaire for COPD Patients) was used to assess the quality of life. A full description of the SGRQ-C version and validation of the studies was published in the journal *Chest* in 2006 [29]. SGRQ-C was developed by Paul W. Jones et al., Division of Cardiac and Vascular Science, St. George’s, University of London [30]. The written consent to its use in the present study was obtained from the author of the questionnaire. The results obtained using SGRQ-C and its subscale, which is divided into three domains: symptoms, activity and psychosocial impact of the disease, enable the determination of the quality of life in the above-mentioned three domains and the general quality of life, and may range from 0 to 100 points, with the result of 0 points meaning the best health/quality of life and minimal impact of the disease on health, while the result of 100 points represents the worst possible quality of life [30]. 

### 2.5. Statistical Analysis

For quantitative variables, the following measures were determined: descriptive statistics: mean values, standard deviation (SD) and value ranges (min–max). For categorical variables, the following measures were determined: number (N) and frequency (%).

Null hypothesis statistical significance testing was used for inference. In order to compare the respiratory function parameter (forced expiratory volume in one second, FEV1) depending on BMI group, Kruskal–Wallis H test with Dunn’s post hoc test was used. Additionally, the correlation between the level of quality of life (SGRQ-C and score of each subscale) and value of FEV1 was analysed. The Pearson correlation coefficient (r) was used to estimate the correlation.

All calculations were performed with STATISTICATM 13.3 software (TIBCO Software, Palo Alto, CA, USA). For all analyses, the *p*-level of <0.05 was considered statistically significant.

### 2.6. Bioethics Committee Approval

The study protocol was approved by the Bioethics Committee at the Medical University of Warsaw.

## 3. Results

### 3.1. Anthropometry

The mean body weight in COPD patients treated with LTOT was 78.2 ± 2 kg, while the mean height was 165.2 ± 9.8. The assessment of the nutritional status depending on the BMI is given in Table 1. The patients were divided into 4 groups: BMI <20 characterized malnourished patients, BMI between 20 and 24.9—patients with normal body weight, BMI between 25 and 29.9—overweight patients and BMI > 30—obese patients.

### 3.2. Spirometry

Table 2 presents the assessment of lung function in patients with COPD treated with LTOT.

**Figure 1:**
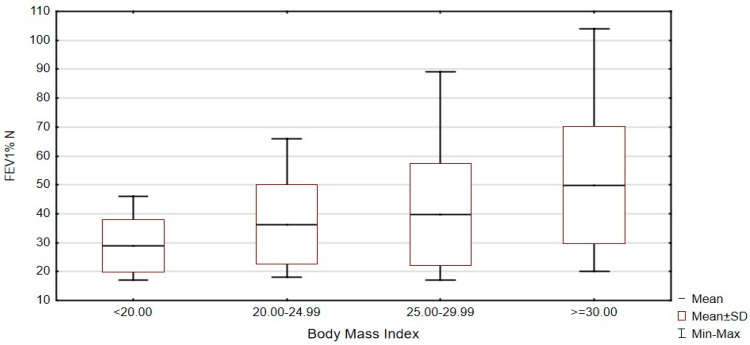
Assessment of the degree of airflow obstruction (FEV1% N) depending on the BMI (BMI < 20.00 BMI 20.00–24.99, BMI 25.00–29.99, BMI > 30.00) in patients treated with LTOT, Kruskal–Wallis test: H = 11.492; *p* = 0.0093, *n* = 110.

When assessing the degree of airflow obstruction (FEV1% N) depending on the BMI in patients treated with LTOT, a statistically significant correlation was noticed between the BMI and the value of the FEV% N parameter (Kruskal–Wallis test: H = 11.492; *p* = 0.0093) (Figure 1). COPD patients with BMI >30 have statistically significantly higher FEV1% N values than patients with BMI in the range of 20–24.9 (Dunn’s test: z = 2.831; *p* = 0.0278). Mean values of FEV1% N in patients treated with LTOT with BMI <20 is 33.29 ± 15.36, with BMI 20–24.9 is 36.6 ± 15.55, with BMI 25–29.9 is 38.7 ± 16.47, and with BMI >30 is 49.94 ± 20.59. 

### 3.3. Nutrition Characteristics of Patients with COPD Treated with LTOT Based on a 3-Day Nutrition Diary

Based on the analysis of the 3-day nutrition diary, the average intake of nutrients, vitamins and minerals in the diet of COPD patients treated with LTOT was calculated (Table 3).

Table 3 presents parameters for the assessment of the diet, dietary intake of nutrients, minerals and vitamins in patients with COPD treated with LTOT based on the calculations made in the Diet 5 program from a 3-day nutrition diary.

In the study group, we observed a weak negative correlation of dietary protein (g) and energy (kcal) consumption with lung function indices: FEV1% N (r = −0.29, *p* < 0.05 and r = −0.25, *p* < 0.05) and MEF 50 (L/s) (r = −0.31, *p* < 0.05, r = −0.27, *p* < 0.05) and a negative correlation between dietary sodium intake (r = −0.36, *p* < 0.05), iron (r = −0.23, *p* < 0.05), zinc (r = −0.28, *p* < 0.05), vitamin B12 (r = −0.31, *p* < 0.05) and the FEV1% N. There was no correlation between vitamin C intake and parameters measured by spirometry, and between dietary intake of individual nutrients and quality of life parameters. 

### 3.4. Characteristics of the Quality of Life in COPD Patients Treated with Long-Term Oxygen Therapy

The overall result of the St. George Questionnaire in patients in the study group and the results of its three components, i.e., symptoms, activity and impact on life, are presented in Table 4. 

In the group of patients, a correlation was observed between the domain of the SGRQ-C questionnaire—activity—and the FEV1 (litres) (r = −0.24, *p* < 0.05); no correlation was observed between other domains of quality of life and general quality of life, and the FEV1 (litres). The mean values of the FEV1/FVC (%) negatively correlated with the general quality of life and the mean values of impact on life (r = −0.24, *p* < 0.05) in the study group. 

## 4. Discussion

The number of studies on the diet of COPD patients treated with long-term oxygen therapy is negligible and therefore difficult to compare. In Poland, it is the first study to assess diet and consumption of individual nutrients, minerals and vitamins in COPD patients treated with long-term oxygen therapy.

In the study group, it was shown that the greatest number of COPD patients treated with long-term oxygen therapy was diagnosed with obesity, based on the BMI index; it affected 34.6% of patients in the study group. Additionally, in the study group, patients with a higher BMI value have lower airflow obstruction, measured as FEV1% N.

Many studies have shown abnormal nutritional status in COPD patients and they are the most common extrapulmonary symptoms in this group. In patients hospitalized due to exacerbations, a positive correlation was demonstrated between body weight and FEV1, and a negative correlation was found between BMI and the duration of hospitalization [15]. The correlation between the BMI and mortality appears to vary with the severity of COPD. A meta-analysis of 22 studies that included 21,150 COPD patients found that, compared to patients with a normal BMI, underweight patients had higher mortality (relative risk (RR) = 1.34) compared with overweight patients (relative risk (RR = 0.47) and obese patients (relative risk (RR) = 0.59). The conclusion was drawn that being overweight and obese acted somewhat as “protection” against death, and that in patients with a normal BMI and underweight patients, the risk of death was greater [28]. This lower risk of death in overweight and obese COPD patients has been called the “obesity paradox” [31]. In patients hospitalized due to exacerbations, a positive correlation was demonstrated between body weight and FEV1, and a negative correlation was found between the BMI index and the duration of hospitalization [32].

Although its mechanism is not fully understood yet, the obesity paradox seems to be partly related to the presence of greater fat-free body mass (FFM) in these patients [33]. Given that FFM and exercise capacity are independent predictors of death, and that respiratory muscle strength is related to performance, these results may at least partially explain why overweight or obese COPD patients often have a higher survival rate [34]. Until now, the nutritional status of patients treated with LTOT has been assessed in a small number of studies. It seems that there is a need for more studies on the nutritional status and lung function in patients treated with LTOT in order to compare and evaluate the obtained parameters.

In the study group, in 51.8% of patients, the average energy consumption was lower than the recommended standards, and in 37.3% it was higher. More than half of the COPD patients did not meet their energy needs in their daily diet. Several factors may explain the low energy intake in COPD patients. Firstly, the most common systemic symptoms of COPD, such as dyspnoea, fatigue and anxiety, may suppress appetite and reduce energy consumption, and some pro-inflammatory cytokines may exacerbate anorexia [5,34,35,36]. Secondly, local factors such as oral diseases, tooth loss, dry mouth and oesophagus, which are common in COPD patients, may contribute to a reduction in energy supply and dietary deficiencies [37].

On the other hand, almost 33.3% of the study participants did not meet the standard of protein consumption for the Polish population, not to mention the increase in its demand, as suggested by studies in patients with reduced fat-free body mass [38]. Interestingly, in the largest percentage of COPD patients (67.8%), dietary fat intake was normal according to the recommendations of the Institute of Food and Nutrition [22]. Low protein intake with excessive or normal fat intake could lead to sarcopenic obesity [5,38,39]. In addition, in COPD patients, lower energy intake (accompanied by increased resting energy expenditure) and unbalanced macronutrient intake (e.g., low protein content) compared to healthy control groups [5], mainly in the presence of obesity [40], suggesting increased risk of malnutrition and the associated adverse consequences in COPD, have been documented.

We obtained negative correlations between the number of calories and protein consumed, FEV1% N scores and MEF 50 values. These correlations indicate that patients on a high-calorie, high-protein diet also have poorer pulmonary indexes. It should be noted that the study group consisted of patients with very diverse nutritional status assessments. Most patients were diagnosed with obesity; however, obese patients may also be patients diagnosed with malnutrition. The causes of the abnormal nutritional status in COPD are considered to be multifactorial and the mechanisms of their development are not yet fully understood. Further research is needed on nutritional assessment also evaluating body composition, diet and its influence on lung function in patients treated with long-term oxygen therapy in order to compare the results of our study with the results of subsequent studies.

It has long been known that an adequate supply of calcium (actually calcium salts) plays a key role in the development of the skeletal system and prevents bone loss. The consumption of calcium salt in 95.4% of COPD patients was lower than that recommended for women and men [22]. Additionally, in the study by Yilmaz et al. [41], the group of COPD patients did not ensure the daily calcium intake in 92.3% of patients. The average calcium intake in the studied COPD patients was 474 ± 311.3 mg and was much lower than in the study by Yilmaz et al. [41], where the average calcium intake was 740.2 ± 310 mg. Insufficient dietary calcium intake of the studied COPD patients may increase the risk of osteoporosis, worsen the prognosis and adversely affect functioning of the respiratory system. The average intakes of vitamin D in COPD patients was very low. The consumption was lower than recommended in as many as 95.5% of COPD patients treated with long-term oxygen therapy. However, in a study by Laudisio et al. [5], over 75% of COPD patients had deficiencies in dietary vitamin D. As proven by Moberg et al. [42] and Yumrutepe et al. [43], reduced intake of vitamin D is associated with poorer lung and muscle function in COPD patients, a greater risk of osteoporosis, atherosclerosis, insulin resistance and a negative impact on the immune system. Our study showed no correlation between vitamin D intake and lung function in patients treated with long-term oxygen therapy.

Ferreira et al. [44] suggested that higher dietary intake of vitamin C and a higher serum concentration of vitamin C protect against respiratory symptoms, while in studied COPD patients, in 78.2% of patients, there were dietary deficiencies of vitamin C. In the study by Pirabbasi E et al. [45], assessing the concentration of individual antioxidants in the blood plasma and in the diet of COPD patients, vitamin C intake was insufficient in 94% of them. In the study group, there was no correlation between dietary vitamin C intake and the parameters measured by spirometry, assessing the respiratory system. 

In the studied COPD patients, a poor-quality diet is also evidenced by high magnesium deficiency, because as much as 83.8% of COPD patients had less than the recommended intake. Early population-based studies reported a strong correlation between magnesium intake and lung function, airway hyper-responsiveness and wheeze [46]. More recently, in a general UK population cohort, intake of magnesium was cross-sectionally related to higher FEV1 (a 100 mg/day higher magnesium intake was associated with a 52.9 mL higher FEV1 (95% CI, 9.6–96.2)), but no correlation between intake of magnesium and longitudinal decline in FEV1 was seen [47].

We also found a negative correlation for sodium, which is an indicator of salt intake in the daily diet. In a study conducted by Hirayama et al. [48], it was found that dietary sodium intake is higher in COPD patients compared to healthy control groups and is associated with lower lung function. Highly processed foods often contain high amounts of sodium.

There was also a correlation between the consumption of iron and vitamin B12, dietary components, of which good sources are, among others, animal products, mainly red meat and cold cuts, and the FEV1% N scores. Greater dietary consumption of iron and vitamin B12 was associated with greater airflow obstruction in the respiratory tract. We believe that this may be related to patients’ consumption of large amounts of red meat and processed meat products, which are potentially harmful to lung function and COPD.

Increased consumption of cold cuts was independently associated with the obstructive spirometric pattern in a cross-sectional analysis of the third National Health Study and Nutrition Examination Survey [13] and with an increased risk of newly diagnosed COPD in both men and women in prospective US cohorts, regardless of the Western nutritional pattern (with great amounts of red meat) or other related foods (refined grains, desserts, etc.). As summarised in a recent meta-analysis, available evidence indicated a 40% increased risk of COPD with higher consumption of processed red meat (>75–785.5 g/week) [49]. This data suggests that pro-health efforts should include specific advice on reducing red/processed meat consumption. 

The general quality of life of patients was about 70% worse as a result of COPD. Activity was limited most and this deteriorated the patients’ quality of life to the greatest extent. The smallest impact on the deterioration of the quality of life, assessing the three domains of the questionnaire and the general quality of life, was obtained in the impact of the disease on patients’ functioning, i.e., the overall impact of the disease on life. It was also noticed that the worse the activity assessed using the SGRQ-C questionnaire, the greater the airflow obstruction in the respiratory tract in the study group of patients receiving long-term oxygen therapy.

More animal experiments and human intervention studies are needed to confirm the effectiveness and mechanisms of diet in preventing and treating COPD.

Although the current nutritional guidelines for the treatment of COPD do not formally provide specific dietary recommendations other than nutritional counselling for malnourished patients, the available scientific evidence provides new directions for future research to justify the role of nutrition in lung function maintenance, respiratory disease prevention and treatment, leading to definitive positioning of nutrition on the roadmap for optimal respiratory health. We believe that knowledge of the effects of diet on COPD can provide healthcare professionals with the correct lifestyle approach to better advise patients on improving their lung health.

## 5. Conclusions

The diet of COPD patients treated with long-term oxygen therapy was improperly balanced and was characterized by significant nutritional deficiencies. The intake of calcium, vitamins A, C, D, E, and folates was lower than the recommended daily intake in over 95% of COPD patients treated with long-term oxygen therapy. Airflow obstruction was significantly lower in obese patients, and greater in patients diagnosed with malnutrition. The general quality of life of patients was approx. 70% worse as a result of COPD. The greatest number of COPD patients treated with long-term oxygen therapy were diagnosed with obesity. 

### Study Limitations

This study has some limitations. The first limitation is a small number of patients with BMI < 20—only 9 patients. Another limitation is the failure to perform blood tests to assess nutritional status. Finally, the study might be enhanced with a control group. However, this should be done by the propensity score matching. We tried to obtain a reliable control group in such a way, but it was impossible. Nevertheless, we plan to continue our research and assess the nutritional status of patients more precisely.

## Figures and Tables

**Table 1:** Assessment of the nutritional status of patients with COPD treated with LTOT according to the Body Mass Index (*n* = 110).

	LTOT (*n* = 110)	
	*n*	%
BMI < 20	9	7.5
BMI 20–24.9	31	28.4
BMI 25–29.9	32	29.3
BMI > 30	38	34.8

**Table 2:** Assessment of lung function in COPD patients (*n* = 110).

Parameter		LTOT
FEV1 FVC (%)	Range	27.9–69
	M ± SD	53.9 ± 12.5
FEV1 (L)	Range	0.3–2.7
	M ± SD	1.0 ± 0.4
FEV1 (% normal range)	Range	17–104
	M ± SD	40.9 ± 18.3
VC (L)	Range	0.5–3.73
	M ± SD	2.1 ± 0.7
VC (% normal range)	Range	28–119
	M ± SD	64.5 ± 18.6
MEF 50 (L/s)	Range	0.1–4.1
	M ± SD	0.6 ± 0.6
MEF 50 (% normal range)	Range	4–83
	M ± SD	17.3 ± 15.2

FEV1—Forced Expiratory Volume in One Second, VC—Vital Capacity, FVC—Forced Vital Capacity, MEF50—Maximal Expiratory Flow at 50% of FVC.

**Table 3:** Average consumption of macronutrients, vitamins and minerals and the percentage of patients with COPD treated with LTOT, who consumed an adequate amount of nutrients, an amount of nutrients below the recommended norm of consumption, and consumed the amount of nutrients above the recommended norm of consumption for the Polish population (*n* = 110) [22].

Parameter	Actual Consumption	Recommended	Consumption	(*n* %) ^1^
	Mean ± SD	<Norms	Norm	>Norms
Energy (kcal)	1906.7 ± 822.5	–	–	–
Protein (g)	77.1 ± 36.4	–	–	–
Fat (g)	68.2 ± 33.7	–	–	–
Carbohydrates (g)	263.6 ± 119.8	–	–	–
% protein	15.6 ± 4.3	33.3	49.4	17.2
% fat	30.9 ± 8	3.4	67.8	28.7
% carbohydrates	53.5 ± 9.3	37.9	59.7	2.3
Sodium (mg)	2172.2 ± 1063.7	58.6	26.4	14.9
Potassium (mg)	2953.5 ± 1263.7	58.6	9.2	32.2
Calcium (mg)	474 ± 311.3	95.4	0	4.6
Phosphorus (mg)	1107.9 ± 465.8	13.8	2.3	83.9
Magnesium (mg)	250.7 ± 98.9	83.9	9.2	6.9
Iron (mg)	10.5 ± 4.2	37.9	20.6	41.4
Zinc (mg)	10.1 ± 3.9	39.1	21.8	39.1
Copper (mg)	1 ± 0.4	29.8	19.5	50.6
Vitamin A (µg)	718.3 ± 608.5	60.7	9.2	29.8
Vitamin D (µg)	4 ± 9.6	95.5	2.3	2.3
Vitamin E (mg)	6.9 ± 4.5	72.4	9.2	18.4
Vitamin B1 (mg)	1.2 ± 0.6	50.6	13.7	45.8
Vitamin B2 (mg)	1.4 ± 0.6	36.7	16.1	47.2
Vitamin B6 (mg)	1.8 ± 0.8	31	22	47
Folate (µg)	179.5 ± 87.5	96.6	2.3	1.1
Vitamin B12 (µg)	4.4 ± 6.7	37.9	52.8	9.2
Vitamin C (mg)	53.3 ± 57.8	78.2	1.1	14.9

^1^ Percentage of Recommended Dietary Allowance (RDA), Appropriate Intake (AI) and Tolerable Upper Intake Level (UL) for adult age/gender groups [22].

**Table 4:** Characteristics of the quality of life in patients in the study group.

Parameter SGRQ		LTOT
(Points)		(*n* = 110)
	M ± SD	64 ± 19.5
Symptoms	Range	11.6–100
	M ± SD	80.2 ± 19.2
Activity	Range	22–100
	M ± SD	63.1 ± 19.7
Impact on life	Range	16.8–99.9
	M ± SD	68.5 ± 16.6
Total	Range	23.8–9.9

## Appendix A

In all study participants, body weight, expressed in kilograms, was measured using a legalized scale with a reading accuracy (d) of up to 20/50 g (WPT 60/150W Personal Scale). During the examination, the patients were barefoot and wore light clothing. Body height, expressed in centimetres, was measured using a stadiometer (WPT 60/150W Personal Scale) as the distance from the base to the highest anatomical point on the head (Vertex). Based on the obtained results of height and weight measurements, the Body Mass Index (BMI) was calculated according to the formula: BMI = body weight (kg)/body height (m^2^). The BMI was categorized into four groups according to the recommendations of the World Health Organization (WHO): (a) malnutrition (18.5 kg/m^2^), (b) normal body weight (18.5–24.9 kg/m^2^), (c) overweight (25.0–29.9 kg/m^2^), (d) obesity (≥30.0 kg/m^2^). The BMI determining malnutrition is controversial, and after reviewing the large number of published studies on the nutritional status of patients with COPD and systemic sclerosis, the limit BMI value was changed to 20 kg/m^2^ due to its most frequent use. In the meta-analysis performed by Chao et al. [28] assessing the correlation between the BMI and mortality in COPD patients, including 22 studies conducted among 21,500 patients, the cut-off value most often defining malnutrition was 20 kg/m^2^.
